# Data Science in Medical and Healthcare: Current Landscape

**DOI:** 10.14789/ejmj.JMJ24-0037-R

**Published:** 2025-04-10

**Authors:** WATARU UCHIDA, GUO SEN, SUN ZHE, TIANXIANG LYU, CHRISTINA ANDICA, KAITO TAKABAYASHI, KEITA TOKUDA, KEIGO SHIMOJI, KOJI KAMAGATA, YOSHITAKA MASUTANI, MITSUHISA SATO, RYUTARO HIMENO, SHIGEKI AOKI

**Affiliations:** 1 Faculty of Health Data Science, Juntendo University, Chiba, Japan; 1 Faculty of Health Data Science, Juntendo University, Chiba, Japan; 2 Graduate School of Medicine, Juntendo University, Tokyo, Japan; 2 Graduate School of Medicine, Juntendo University, Tokyo, Japan; 3 Department of Radiology, Juntendo University, Tokyo, Japan; 3 Department of Radiology, Juntendo University, Tokyo, Japan; 4 Tohoku University School of Medicine, Miyagi, Japan; 4 Tohoku University School of Medicine, Miyagi, Japan

**Keywords:** health data science, magnetic resonance imaging, supercomputer, synQSL, brain simulation

## Abstract

Data science is revolutionizing various industries and its impact on healthcare and life sciences is particularly profound. The vast amounts of data generated in these fields present both opportunities and challenges, necessitating professionals to extract insights and create value from these data resources. However, effective data-driven solutions in healthcare require a unique combination of technical data science skills and deep-domain expertise in areas such as medicine, public health, and sports science. This review discusses the growing importance of domain knowledge in data science and the need for interdisciplinary professionals who can bridge the gap between data analysis and practical applications in the healthcare sector. Furthermore, this paper highlights specific applications of data science in healthcare and life sciences, leveraging artificial intelligence (AI) and advanced computational methods. By integrating cutting-edge data science techniques with profound domain understanding, these applications aim to drive innovation, advance medical research, improve patient outcomes, and deepen our understanding of human health and well-being. Overall, this review underscores the synergies between data science and domain expertise in healthcare and life sciences, emphasizing the importance of interdisciplinary collaboration in unlocking the full potential of data-driven solutions in these critical fields.

## Introduction

Data science is an interdisciplinary field that combines statistical techniques, computer science, and domain expertise to extract insights and create value from large amounts of data. In an era of information overload, data science plays a critical role in transforming raw data into actionable knowledge, enabling informed decision-making and driving innovation across industries^1-3)^. At its core, data science involves the collection, processing, and analysis of data using advanced computational methods and algorithms. This process includes data mining, machine learning, artificial intelligence (AI), statistical analysis, and data visualization techniques^3)^. However, data science goes beyond mere computational analysis; it emphasizes the importance of domain knowledge and collaboration with experts to interpret results and ensure their practical applicability and societal impact. Moreover, data science is not limited to a specific industry or domain; rather, it is a powerful tool that can be applied to diverse fields, including medicine, social sciences, marketing, and the humanities^4)^. In particular, in medicine and healthcare, there have been attempts to apply data science to create value in the diagnosis and treatment of diseases and the promotion of health^4)^. However, effective data-driven solutions in healthcare and life sciences require a deep understanding of both the technical aspects of data science and the domain knowledge^5, 6)^. This review presents the growing demand for people with specific domain knowledge in the modern era, discuss the prospects and challenges of data science in the health sciences, and highlights specific applications that combine medical data with advanced data science techniques.

## Is the domain knowledge in a specific field important for a data scientist?

In today’s data-driven world, the amount of data is staggering and continues to grow exponentially. According to a report by the International Data Corporation (IDC), the global dataset is expected to grow from 33 zettabytes (ZB) in 2018 to 175 ZB by 2025^7)^. This deluge of data presents both opportunities and challenges, increasing the demand for professionals who can extract insights from specific fields and create value from these vast data resources. Despite the surge in demand, there is a significant gap in the education of data analytics and domain knowledge in diverse fields. Traditional data science programs often focus primarily on technical skills such as programming, statistics, and machine learning algorithms^6)^. However, as data science applications become more widespread across various domains, there is a growing need for professionals with a combination of diverse analytical skills and domain- specific expertise^5, 6)^. The Business-Higher Education Forum (BHEF), a forum organized by about 55 top leaders of U.S. businesses, universities, and museums, reports that job postings seeking data science skills coupled with industry or domain knowledge increased by 90% between 2015 and 2018^8)^. This trend underscores the importance of domain knowledge in delivering effective and contextually relevant solutions. That is, mere technical proficiency in data analysis and modeling might be insufficient; the ability to interpret results, identify relevant patterns, and translate insights into actionable strategies necessitates thorough comprehension of the domain in question. Recognizing this need, several universities have established new programs or faculties aimed at cultivating professionals with interdisciplinary knowledge that spans data science and specific domains^6)^. Focusing on the medical and healthcare field, for example, Harvard University launched the Master of Science in Health Data Science program, offering curricula to address questions in public health and biomedical sciences. In Japan, new programs and faculties have also been established to address the talent gap and meet the growing demand for data scientists with domain expertise. For example, Juntendo University established the Faculty of Health Data Science, aiming to cultivate a new generation of professionals who possess a unique combination of data science skills and domain expertise in fields such as medicine, sports science, and statistics. As data continues to pervade every aspect of society, fostering a workforce with a unique combination of data science skills and domain expertise will become increasingly crucial. The following sections discuss points to keep in mind for a better understanding of medical and health science data.

## Considerations in the medical and health sciences data science

### Data characteristics in medical and healthcare

In recent years, data in the medical and health science fields have been collected from a variety of sources with different characteristics, forming big data with enormous volume, generation speed, and diversity^9)^. The concept of big data is continually evolving, and currently, it emphasizes the process of deriving value from this data^10)^. Data in the medical and healthcare fields are diverse, reflecting the complexity and breadth of the sector. These can be categorized as follows1^1, 12)^:

• Clinical and Health Records Data: This category encompasses electronic health records, patient histories, and clinical documentation, including health histories of various conditions, lifestyle factors, vital signs, therapy procedures, medication records, and allergy information. It also covers treatment outcomes and ongoing health monitoring data.

• Imaging Data: Includes modalities such as magnetic resonance imaging (MRI), computed tomography (CT), positron emission tomography (PET), ultrasound, and X-ray.

• Biomedical and Laboratory Data: This includes a wide range of biological specimens and their analyses. It encompasses comprehensive body fluid analyses including blood tests (complete blood count, biochemistry, immunological markers), cerebrospinal fluid examination, urinalysis, and saliva testing. The category also includes tissue samples, genetic test results, metabolic profiling, and specialized laboratory tests such as drug screenings and disease-specific diagnostic tests.

• Patient-Generated Data: Includes lifestyle information (e.g., exercise, sleep, diet) and self-monitoring data from devices such as glucose monitors and heart rate trackers.

As data sources continue to proliferate in both variety and magnitude, sophisticated analytical approaches are required to swiftly and precisely combine and examine the immense troves of heterogeneous data previously described^13, 14)^. On the other hand, the sheer volume and diversity of these medical data pose a variety of inherent challenges^15)^. Examples of the most common challenges include the creation of an environment for processing large data sources with advanced analytical techniques, data structure (medical data is generally dispersed among and within facilities and is technically sophisticated to aggregate and analyze), ensuring security and privacy, trade-off between costs and quality, and ensuring data storage and transfer environments. This section focuses on the unique challenges that exist in big data analysis using large volumes of medical images and discusses approaches to overcome these challenges.

### Challenging in big imaging data analysis in medical and healthcare

A wide variety of benefits have been increasingly recognized from advanced analysis of vast medical imaging data sets, including application of advanced AI and statistical models, and the multivariate analysis to integrate and evaluate diverse amounts of information. The potential benefits of advanced analysis include improved medical services and increased efficiency and accuracy of various processes such as diagnosis, treatment, and testing by healthcare professionals^11)^. Specifically, for example, an advanced imaging analysis might lead to to diagnostic assistance by automatic detection of lung nodules^16)^ and significant stenosis in the coronary arteries^17)^ using a deep learning segmentation model, and inter-disease classification and prediction of neurodegenerative disorders using MRI^18)^. However, one of the major challenges in the analysis of medical image big data is the enormous size of individual image data and the high rate of data growth acceleration associated with advances in imaging technology. Each image may contain millions of pixels, and the volume of data continues to grow as image processing technology improves and expands, and data acquisition speed increases^19)^. In addition, the importance of multivariate approaches across multiple modalities has been emphasized, leading researchers to acquire more types of images for each subject. Thus, the accumulation of medical imaging data is increasingly becoming a flood, and its computation requires powerful hardware and optimized algorithms for timely analysis and diagnosis^20)^.

The next chapter focuses on MRI technology, the author’s area of expertise, and introduces topics related to the latest AI technologies and the high-speed processing for big data of MRI and their applications.

## Applications in big data of MRI

### Development in large-scale database

In recent years, with the recent expansion of public databases containing thousands to tens of thousands of human brain MRIs, there are increasing expectations for the development of brain image analysis research to elucidate pathological changes underlying the development of neurological and psychiatric diseases and to develop new biomarkers to detect disease progression. Big databases such as the UK Biobank^21)^, Human Connectome Project (HCP)^22)^ and the Alzheimer’s Disease Neuroimaging Initiative^23)^ are invaluable sources of information for medical research. The UK Biobank utilizes data from over 500,000 participants to analyze various disease risk factors and health impacts extensively. Meanwhile, ADNI tracks the progression of Alzheimer’s disease, contributing to early diagnosis and the development of new treatments through detailed data from over 3,000 participants. By combining large-scale databases with complex analytical techniques, we can provide new medical insights. However, the time required for image analysis has increased proportionally and exceeds the amount of analysis possible at a single laboratory level. In particular, brain image analysis often requires 5 to 6 hours per person^24)^ for a series of processes such as segmentation of local regions and modeling of estimated neural microstructure based on the water molecule diffusion using diffusion MRI, and the development of efficient analysis methods is an urgent issue. We attempt to address this issue by using deep learning methods and supercomputers to parallelize massive processing, which is outlined in this review.

### Acceleration for free-water imaging estimation using synthetic q-space learning

Diffusion MRI is a powerful technique that captures three-dimensional Brownian motion changes and models neural microstructural changes by fitting biophysical models. One such advanced model is free-water imaging, which was developed to address the issue of extracellular free-water compartments within a single voxel in diffusion tensor imaging (DTI)^25)^. Free-water imaging aims to model the diffusion of water molecules originating from neural cells by incorporating the free-water compartment into the model^26)^. This technology has been successfully applied to a variety of neurodegenerative, psychiatric, and lifestyle-related diseases, providing insights into the underlying pathophysiological processes^25, 27)-29)^. Despite its potential, the time-consuming fitting process hinders its clinical application. To overcome this challenge, we explored the use of synthetic q-space learning (synQSL)^30, 31)^, a deep learning-based approach with synthetic data based on signal model equations and noise simulation to accelerate free-water imaging. While the output values of simple QSL training data are essentially provided by conventional inference methods, such as fitting, the synQSL method has the advantage that the training data can be synthesized by the simulation process of DWI acquisition. In other words, synQSL enables faster estimation of DWI metrics through deep learning-based parameter inference as well as robust parameter estimation that does not require large and extensive training samples through simulation-based training data synthesis^32)^.

**Figure 1:**
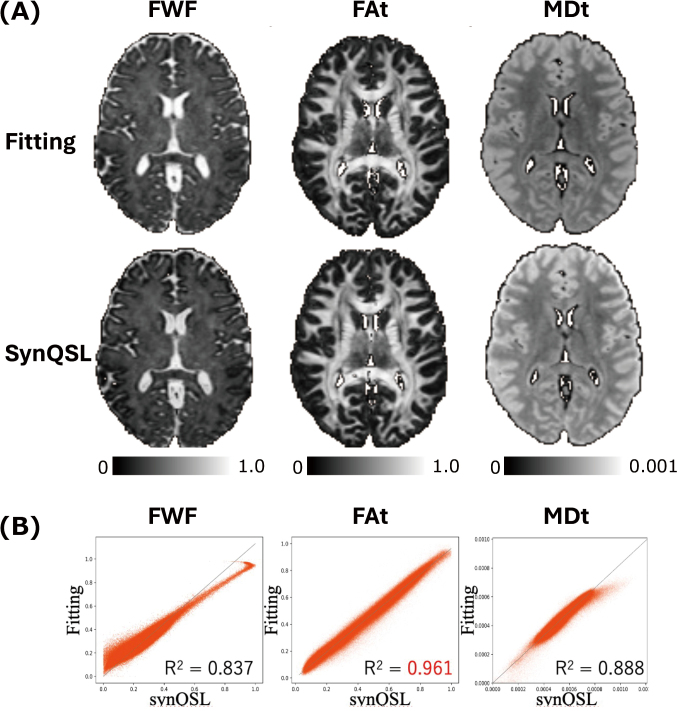
Comparison of free-water imaging parameters estimated by the conventional fitting and calculated based on the synQSL (A) The top panel shows the conventional fitted results and the bottom panel shows our proposed synQSL-based estimation results. (B) Correlation of free-water imaging signal values based on synQSL-based and fitting-based approaches. Abbreviations: FWF, free-water volume fraction; FAt, free-water corrected fractional anisotropy; MDt, free-water corrected mean diffusivity.

Figure 1 (A) shows that there is little difference between free-water imaging parameters estimated by the conventional fitting and by the synQSL visually. In addition, the synQSL-based parameters have also provided strongly correlated values with conventional fitting-based metrics (Figure 1 [B]). This indicates that free-water imaging parameter estimation using synQSL is almost equivalent to conventional fitting. Remarkably, while conventional fitting approaches required approximately 30 hours per case, our proposed method utilizing synQSL accomplished the estimation in only 10 minutes. Namely, our study demonstrates that our approach can estimate the parameters for free-water imaging significantly faster than traditional fitting-based estimations. These remarkable improvements in computational speed might provide tools for comprehensive neuroimaging studies leveraging large-scale datasets, as well as overcoming the computational time bottleneck that has hindered clinical adoption.

### High performance computing driven brain image analysis

**Figure 2:**
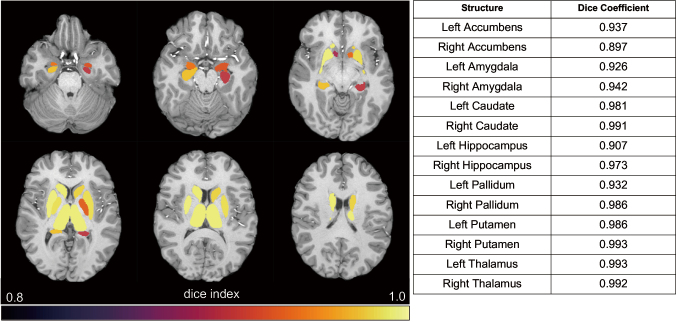
Dice coefficients of deep gray matter segmentation performed on Fugaku supercomputer and conventional node High Dice coefficients for all deep gray matter regions suggest high reliability in high performance computing system. Adapted with permission from Lyu et al^37)^.

MRI data is critical for various neuroscience research applications, including the assessment of macrostructural, microstructural, and network-level brain features in both healthy and diseased states. Large-scale neuroimaging datasets, such as the Human Connectome Project, the UK Biobank, and the Adolescent Brain Cognitive Development study, have been instrumental in enhancing data diversity, statistical power, and robustness of findings. However, processing such extensive datasets involves complex tasks like data correction, brain tissue parcellation, quantitative mapping, brain network construction, and statistical analysis, all of which are computationally intensive and time-consuming. Processing these vast datasets on conventional workstations is impractical, necessitating the use of advanced high-performance computing (HPC) systems. Our study demonstrated the application of HPC, specifically the Fugaku supercomputing system, to accelerate MRI data processing. The Fugaku, a homogeneous, CPU-based distributed machine with 158,976 nodes, uses ARM v8.2A architecture CPUs. This supercomputer supports high-speed data processing with a memory bandwidth of 1024 GB/s and is optimized for running complex imaging processing pipelines efficiently^24)^. The Fugaku supercomputer, a state-of-the-art CPU-based distributed machine, was employed to run the FMRIB Software Library (FSL) on T1-weighted images and diffusion MRIs from the HCP dataset. This approach significantly reduced processing times while maintaining high reliability and consistency in the results. Our approach employed data parallel processing strategies on supercomputers to handle large numbers of brain images. Techniques such as NUMA-aware optimizations and bulk job scheduling were used to maximize the efficiency and speed of the computations. The use of supercomputers like Fugaku and Hokusai has significantly enhanced our ability to process large-scale MRI datasets rapidly and accurately (Figure 2). This advancement not only facilitates our current research but also opens new possibilities for future studies involving even larger datasets from sources such as the UK Biobank. By leveraging HPC, we aim to address the computational challenges posed by massive neuroimaging datasets, which have the potential to accelerate discoveries in brain research. While these advancements may contribute to an improved understanding of neurological conditions and inform future treatment strategies, current limitations in the availability of comprehensive datasets mean that further research and data collection are necessary to realize these possibilities fully.

### Brain simulation

Brain simulation has emerged as a critical field for understanding the complex mechanisms of the brain and addressing neurological disorders. Worldwide, major initiatives are making significant strides in this area:

The Human Brain Project (HBP): Funded by the European Union, this ambitious project aims to create a comprehensive, multi-scale model of the human brain by integrating neuroscience, computing, and data science^33)^.

The United States’ BRAIN Initiative: This initiative emphasizes developing innovative technologies to map and understand brain circuits^34)^.

Japan’s Brain/MINDS Project: This project integrates diverse datasets, including structural, functional, and connectome data, to develop precise simulations of neural networks^35)^.

Brain data analysis studies utilize brain data from large-scale neural activity measurements, whole-brain connectivity measurements, and large- scale brain simulations to reveal the brain’s representation model, expressed as a low-dimensional manifold, and its hierarchical structure of connections and hub structures.

At Juntendo University, we have already developed a data-driven whole-brain model that includes the cerebral cortex, basal ganglia, cerebellum, and thalamus, and we are currently working on a connectome data-driven simulation framework^36)^. In the research, we are leveraging the capabilities of diffusion MRI to obtain detailed structural information of the brain. This comprehensive dataset includes both patient and healthy control data, allowing us to analyze a wide range of brain conditions. By using diffusion MRI, we can map the intricate network of neural connections, providing a high-resolution view of the brain’s architecture. We employ the brain simulation framework in conjunction with the brain connectome data derived from diffusion MRI. This combination enables us to simulate brain activity with a high degree of accuracy, facilitating the exploration of various brain pathologies^24)^. The inclusion of patient data allows us to create specific models that reflect the structural abnormalities associated with different neurological disorders^37)^.

Through these simulations, we aim to gain deeper insights into the mechanisms underlying various brain diseases. By comparing the brain activity patterns of patients and healthy individuals, we can identify potential biomarkers and pathways involved in disease progression. This research not only enhances our understanding of brain function but also has the potential to inform the development of new diagnostic tools and therapeutic strategies for neurological disorders.

Looking ahead, we aim to advance our connectome data-driven simulation framework by incorporating multimodal datasets, including diffusion MRI data and electrophysiological recordings. A significant milestone in our research journey is our recent inclusion in the Brain/MINDS 2.0 project in September this year. Within this initiative, we are collaborating with leading institutions to explore the brain’s hierarchical organization and functional hubs using state-of-the-art simulation techniques. By utilizing high-performance computing platforms such as the Fugaku supercomputer, we plan to enhance the accuracy and scalability of our models, enabling deeper insights into brain function. Furthermore, we seek to expand the application of these models in predictive diagnostics and personalized treatment planning, aligning with Brain/MINDS 2.0’s goals of advancing neuroscience research and its clinical applications. Through this collaboration, Juntendo University is poised to make significant contributions to understanding brain mechanisms and developing innovative approaches to tackle neurological disorders.

## Conclusion

We articulate the profound implications of integrating data science with domain expertise in enhancing healthcare outcomes. We advocate for a symbiotic relationship between technical prowess in data science and nuanced understanding of medical sciences. This interdisciplinary approach is essential for translating vast data landscapes into actionable healthcare insights, thereby revolutionizing patient care and medical research. The conclusion emphasizes the necessity of nurturing professionals who are adept at navigating both the complexities of advanced computational techniques and the intricate details of medical science. This integration promises not only to improve diagnostic and therapeutic precision but also to drive significant advancements in understanding complex health conditions. Through fostering such cross-disciplinary collaboration, the potential of data science in healthcare can be fully realized, leading to more personalized, efficient, and impactful health interventions.

## Funding

This study was supported by the Juntendo Research Branding Project, the Project for Training Experts in Statistical Sciences; the Japan Society for the Promotion of Science Grants-in-Aid for Scientific Research (KAKENHI; grant Nos. 23H02865 and 23K14927), a Grant-in-Aid for Special Research in Subsidies for ordinary expenses of private schools from The Promotion and Mutual Aid Corporation for Private Schools of Japan, and the Brain/MINDS Beyond program of the Japan Agency for Medical Research and Development (grant Nos. JP18dm 0307004 and JP19dm0307101), and Agency for Medical Research and Development (grant No. JP21wm0425006). This study was also supported by the Otsuka Toshimi Scholarship Foundation.

## Author contributions

The first draft of this manuscript was written by WU, GS, and SZ, and all authors commented and revised on subsequent versions. All authors read and approved the manuscript.

## Conflicts of interest statement

The authors declare that the research was conducted in the absence of any commercial or financial relationships that could be construed as a potential conflict of interest.
